# Fostering caring relationships: Suggestions to rethink liberal perspectives on the ethics of newborn screening

**DOI:** 10.1111/bioe.12425

**Published:** 2018-02-14

**Authors:** Simone van der Burg, Anke Oerlemans

**Keywords:** care, ethics, liberalism, newborn screening, technology

## Abstract

Newborn screening (NBS) involves the collection of blood from the heel of a newborn baby and testing it for a list of rare and inheritable disorders. New biochemical screening technologies led to expansions of NBS programs in the first decade of the 21st century. It is expected that they will in time be replaced by genetic sequencing technologies. These developments have raised a lot of ethical debate. We reviewed the ethical literature on NBS, analyzed the issues and values that emerged, and paid particular interest to the type of impacts authors think NBS should have on the lives of children and their families. Our review shows that most authors keep their ethical reflection confined to policy decisions, about for instance (a) the purpose of the program, and (b) its voluntary or mandatory nature. While some authors show appreciation of how NBS information empowers parents to care for their (diseased) children, most authors consider these aspects to be ‘private’ and leave their evaluation up to parents themselves. While this division of moral labor fits with the liberal conviction to leave individuals free to decide how they want to live their private lives, it also silences the ethical debate about these issues. Given the present and future capacity of NBS to offer an abundance of health‐related information, we argue that there is good reason to develop a more substantive perspective to whether and how NBS can contribute to parents’ good care for children.

## 1 INTRODUCTION

Newborn screening (NBS) involves taking a few drops of blood from the heel or pulse of an infant in the first week of life and testing it for a list of serious and treatable diseases.^1^ NBS programs evolved from a biochemical success story: they started from 1963 onwards, when American biochemist Robert Guthrie developed a blood test for phenylketonuria (PKU). PKU is a rare metabolic disease which—if left untreated—results in severe physical and mental retardation. Detection of PKU right after birth, coupled with a strict diet that avoids phenylalanine in food, largely prevented the detrimental effects of the disease. Given this health improvement, and the forceful lobby of pediatricians and parents of PKU‐affected children, many countries in Europe, the Americas, Australia, and Asia started screening all newborn children for PKU.^2^

NBS still targets solely PKU in some countries, but most countries have gradually added more diseases due to increased technological possibilities to detect them.^3^ Expansions of NBS programs in the first decade of the 21st century were largely due to the availability of tandem mass spectrometry, which allows for the detection of over 60 diseases with a single test based on biochemical components of blood. As almost all diseases included in NBS—except for example congenital hypothyroidism, which many programs target—are genetic in nature, it is expected that these biochemical technologies used for screening will be replaced by next generation sequencing (NGS) technologies within 5–10 years, making it possible to disclose an abundance of health‐related information at birth.^4^

These developments have raised a lot of ethical debate. In this article we reviewed this ethical debate on NBS and analyzed the issues and values that emerge. The results of this analysis are presented in Sections 2 and 3 of this article as well as in Table 3. Our analysis will show, however, that the current debate about ethics of NBS has limitations, which can be explained on the basis of its tacit *liberal* presuppositions regarding what are and what are not appropriate topics for debate in policy‐making, including policy about NBS. Consequently, issues about whether and how NBS can help parents to care well for their children are largely ignored, as liberalism prescribes policymakers to refrain from influencing the way in which people choose to lead their private lives including how they raise and care for their children. Many ethicists—especially communitarians and care ethicists—have, however, famously argued in the past that it is impossible to make policy that remains neutral about the good life.^5^ In line with their way of thinking, we argue here that the envisioned future development of NBS programs make it important to discuss whether and how NBS should be allowed to influence caring relationships within the family. In Table 4 we suggest questions that provide a starting point to that debate.

**Table 1:** Categories of diseases or disease information that can be included in NBS

Category of diseases or disease information	Explanation
Treatable diseases	Diseases that can be treated with medication or a diet, preventing or postponing death and significantly improving the prognosis of the child. PKU is the classic example of this disease, as it was the first disease for which most NBS programs started screening.
Untreatable diseases	Diseases for which no treatment is available. There may however be supportive measures available which may improve the condition of the child somewhat. Various metabolic and mitochondrial diseases belong to this category.
Late‐onset diseases	Treatable or untreatable diseases that develop later during childhood, adolescence, or adulthood. Examples are Fabry disease or Huntington's disease.
Risk information	Risk information does not provide the benefit of certainty, but reveals a probability that a person will develop a disease—such as breast or colon cancer, or diabetes—later in life. Sometimes interventions are available to prevent or decrease the probability of the disease occurring; sometimes no such measures are available.
Carrier status information	Carrier status (CS) information is not beneficial to the health of the infant; it will only become (potentially) relevant to the child when it reaches reproductive age. CS information may be a by‐product of screening with biochemical technologies such as tandem mass spectrometry or high‐performance liquid chromatography. However, if WGS will be used to conduct NBS in the future, it will likely lead to the identification of a greater number of carriers of different conditions.
Disease information that is difficult to classify	Some disease information may be difficult to classify in any of the above categories. Duchenne Muscular Dystrophy, for example, is a serious late(r)‐onset disease in boys for it reveals its first symptoms at age 4–5. Girls are only carriers and 80–90% of girls will remain asymptomatic. Lysosomal storage disorders may produce symptoms later in childhood, but patients may also remain symptom‐free until they are middle‐aged. Pompe disease has earlier‐ and later‐onset variants. Including these diseases means that NBS will produce different types of information for different people and some people would receive a diagnosis long before there are symptoms—if symptoms occur at all.

**Table 2:** Categories of results that deviate from the program

Categories of results that deviate from the program	Explanation
False positives	Occurs when parents receive a positive result from NBS that indicates the child is diseased, but after follow‐up diagnostic tests the child turns out not to have the disease after all.
Incidental/unsolicited/unanticipated findings	Findings not targeted by the NBS program which the technology used for screening may accidentally produce. These findings can come in different varieties. They may reveal: the carrier status of an infant for a disease included in the program variants of diseases included in NBS that fall beyond the scope of the program (because they are mild and harmless or serious and untreatable) patients with variants of early‐onset diseases that are later‐onset (e.g., in adulthood) misattributed paternity the mother is diseased instead of the child a disease completely unrelated to diseases included in NBS (in NGS)
Uncertain or ambiguous test results	Findings that are suspect, but are not clearly associated with a disease. These may be: biochemical variants of unknown significance or ‘immature’ genetic variants which have not yet been given a stable interpretation genetic anomalies whose connection to the phenotype is unknown disease with varying penetrance from patient to patient
Overdiagnosis	A biochemical or genetic anomaly related to diseases included in the NBS program, but which does not actually cause disease symptoms in a particular person. Examples of non‐diseases are variants of PKU and short‐chain or medium‐chain acyl CoA dehydrogenase deficiency, duarte variants of galactosemia or 3‐methylcrotonyl‐CoA carboxylase deficiency, which may present in varieties ranging from no symptoms to death.

**Table 3:** Issues in the ethical literature on NBS

Issues	Values
*Relating to the justification of the NBS program*	
• What are the conditions that an NBS program must meet to justify the spending of public money on NBS? • Are inequalities between NBS programs in different countries/states/provinces acceptable? • On what grounds can inequalities between NBS programs be justified? • What goal(s) should NBS serve? • What criteria should be used to justify the selection of diseases/disease information in NBS? • What/whose benefits should be served with NBS? • Should benefits/harms of NBS be understood individually or as family or societal benefits/harms? • In what way should we weigh the different benefits/harms that inclusion of a disease/disease information can produce for different people? • If it is possible to produce disease information with NBS, can it justifiably be withheld from parents? What/whose rights should be served/protected by NBS? • How should findings that exceed the limitations of the NBS program be dealt with? • (How) should NBS professionals prepare parents for findings that exceed the NBS program? • (How) should professionals return findings that exceed the program to parents? And can they justifiably withhold these findings from parents? • How should professionals make responsible decisions, if they are confronted with uncertain findings? How should trust in the NBS practice be maintained in spite of findings that exceed the limitations of the program?	Justice Distributive justice/equality Benefits and harms Hard impacts: health, gained life years; (avoidance of) suffering Soft impacts: wellbeing, healthy psychological development, avoidance of stress/guilt, warm, and supportive bond between parents and children, good care, fostering future autonomy of children, supportive environment, equal opportunity (nonstigmatization; nondiscrimination), being able to plan one's life (Reproductive) autonomy Right to an open future Privacy Right not to know Knowledge, health of future generations Avoidance of medicalization (good care) Stable family life (good care) Acceptance Trust, active participation in NBS
*Relating to the manner in which parents are involved in NBS*	
Should involvement in NBS be mandatory or voluntary? Is it justified to supersede parental autonomy in order to protect the health of children? Should parents’ autonomy be protected, even if it means they can say ‘no’ to NBS and thus (potentially) harm their child? What conditions should an NBS program meet to justify mandatory screening? Can mandatory screening (still) be justified in expanded NBS programs? What conditions should an NBS program meet to justify voluntary screening? Can the preconditions for voluntary screening (still) be met in expanded NBS programs? What model of participation should be adopted in NBS? If it is to protect the health of children? If it is to foster public trust in NBS? If it is to respect autonomy of parents to give shape to their private practice of child bearing, rearing and family?	Autonomy Freedom Protection of health Knowledge/being informed Active participation Honesty Trust Privacy Reproductive autonomy Stable family life (caring bond) Care

**Table 4:** New issues

• Where should the distinction between the public and private domain in NBS be drawn? • How do children, parents and NBS policy depend on each other? • What is good care for children and how can NBS contribute to it? • What is the meaning of relational autonomy/relational personhood in NBS, and what does it imply for the goals of NBS? • What substantive responsibilities do children, parents and NBS policy have toward each other and how do these responsibilities change when NBS expands? • What is the meaning of relational values like care, trust, reciprocity, and solidarity in NBS? • Can/should a liberal perspective on NBS be complemented with a relational or care ethics perspective? • What values does a liberal perspective/relational care ethics perspective allow us to see and appreciate in the further development of NBS? • (When) is it justified to keep available information from parents? • Can NBS policy remain neutral and leave decisions about parenting to parents? • If NBS cannot remain neutral about good parenting, what perspective on ‘good parenting’ should NBS foster? • To what extend does this perspective on ‘good parenting’ coincide with the values of actual parents? • If NBS fosters substantive values about parenting, how should it respond to dissenting parents?

## 2 METHODS

In this paper, we aimed to review the issues covered in ethical debates on NBS. By combining search terms related to these two elements (ethics and NBS), we searched for English language articles published between January 1, 2001 and July 1, 2016 in PubMed. Technologies able to detect many diseases with a single test were introduced in NBS programs starting in 2001, hence the chosen time period.

We screened the titles and abstracts of all articles identified for their eligibility. For inclusion, each paper had to (a) concern NBS for inherited diseases during the first weeks of life, making use of blood withdrawal, (b) contain discussion of ethical issues, and (c) be nonempirical in nature (i.e., theoretical, analytical, argumentative, opinionating, commentaries, conceptual, or review articles). When inclusion or exclusion could not be decided on the basis of title and abstract, we retained and reviewed a full‐text copy.

Our initial search identified 336 records. Based on title and abstract, 193 could be excluded. The remaining 143 papers were read in full. A further 42 were excluded because they were unavailable, were not about NBS, did not contain any discussion of ethical issues, and/or were solely empirical in nature.

After this selection, 101 articles remained. We reviewed the content of these articles, and ordered the issues and questions in themes and subthemes, presented in Tables 1, 2, 3. A full search strategy containing the search terms used as well as a complete record of the reasons for exclusion at each step of the process is available upon request.

## 3 RESULTS

While NBS is a population screening program, it is not common in the ethical literature on NBS to reflect explicitly on the question whether and how this program is allowed to interfere in the lives of children and parents. The ethical discussion about NBS does, however, reveal various presuppositions about family relationships, while it discusses other themes. In this discussion we can distinguish two general themes^6^:

1. The justification of the purpose of NBS, focusing on questions relating to (a) scarcity of healthcare resources, (b) human rights, (c) the choice of diseases included in the NBS panel, and (d) results that deviate from the official program;
2. Whether parental participation in the program ought to be voluntary or mandatory.

### 3.1 The justification of the purpose of the NBS program

#### 3.1.1 A just spending of scarce healthcare resources

The choice of diseases in an NBS program, first, needs to be justified with respect to a balancing of the costs and benefits for society: this discussion concerns justice and focuses on the relationship with the population at large as tax payers and recipients of healthcare services.^7^

Justice is for example discussed with respect to the unequal access to screening or treatment. While different governments can make their own decisions about NBS, the resulting inequality between programs is often considered unjust as it means that children in one country or state are screened for a particular disease and receive treatment, while children born in the next country or state will lack screening and presymptomatic treatment and may therefore develop serious health issues.^8^ Furthermore, it is considered problematic that follow‐up services are not equally available or accessible everywhere^9^; in some contexts treatment for rare diseases included in NBS is not covered by insurance, which means NBS will only produce benefits for relatively wealthy people who can pay for treatment.^10^ In some countries (part of) NBS is privatized, such as in the United States where NBS consists of a mandatory primary list of diseases and a secondary optional one. Participation in screening for this secondary list depends on parents’ selective knowledge and capacity to pay.^11^

Inequality within and/or between countries/states will likely increase when more high‐tech technologies are introduced in NBS. Genomic sequencing technologies for NBS are available only in specialty medical centers and are not always covered by health insurance, thus limiting access to NBS services to those who can pay for them in wealthy countries.^12^ Implementation of these technologies is also costly, as they require tracking systems and training of physicians, nurses, and counselors to inform parents. Not every country will have the resources to cover these additional costs.^13^

Last, authors question whether NBS programs are launched and expanded too quickly without a systematic effort to compare potential benefits of NBS to alternative ways to use public resources to help children.^14^ With respect to NBS in India, Miller argues it is problematic to spend money on NBS because genetic disease offers only a limited contribution to the overall child mortality there.^15^ Moyer et al. criticize the spending of public money on NBS more generally, because its claimed benefits are insufficiently supported by scientific evidence.^16^ It is difficult to provide the required evidence to prove candidates for inclusion in NBS can be detected and treated: these diseases are very rare, come in different variants (from mild to life‐threatening), and the medical contexts in which they are detected and treated differ significantly. Therefore, it is concluded that science alone cannot offer sufficient justification for spending on NBS; values are needed.^17^

#### 3.1.2 Human rights

Human rights and specifically children's rights are a recurring ethical theme in NBS literature. All NBS programs have to respect basic human rights, thereby limiting the way in which NBS can relate to parents and their children. However, the literature focuses only on a small selection of these rights: rights demanding to respect the (future) autonomy of the child and to protect privacy, which isolates children as individuals and reveals they may have interests different from those of their parents, healthcare providers, researchers, or policymakers.

In discussions about the purpose of NBS, human rights function as a way to legitimize the selection of some diseases in the program, and oppose the inclusion of others. The inclusion of treatable diseases, for example, serves the right to an open future for children, as it allows them to grow up as healthy as possible and develop their autonomy as adults. Alternatively, returning information about the risk of developing a disease later in life—during adulthood—does not respect their right to an open future as returning this disease information to parents after birth denies children the chance to decide for themselves when they are adults whether they want to obtain this information.^18^

Furthermore, communicating these types of information at birth is thought to infringe on the child's right to confidentiality of medical information.^19^ Parents may share this information with family members, friends and teachers, and deny their children privacy as well as the possibility to decide for themselves with whom they want to share it.^20^ In addition, parents may be required to report this information, which can lead to discrimination by insurance companies, employers or other parties, complicating a child's future life choices.^21^

Other authors argue that disclosure of information about risk, adult‐onset disease, and carrier status actually serves human rights, as it allows counseling parents about their own carrier status and their risk of having affected children in the future.^22^ It therefore serves the reproductive autonomy of parents. As parents do not always use this information in their reproductive planning, some authors argue that the at‐risk community should be consulted to find out whether they actually value having carrier status information.^23^ Other authors consider it questionable or even ‘ethically worrisome’ to require parents to receive carrier status information, as this may give the impression that NBS aims for the prevention of the birth of these children, which suggests a policy of eugenics.^24^ Provision of this information is considered an intrusion in parents’ private lives and the opinion that these authors defend is that policy should not interfere with that life.

#### 3.1.3 ‘Benefits’ and ‘harms’ produced by NBS

Questions about how much (or how little) NBS may interfere with lives of children and their parents, mostly concern the desirability of NBS’ impacts. Discussions about the desirability of the return of various types of disease information feature primarily consequentialist language, as NBS promotes human welfare and is therefore outcome‐oriented (in terms of ‘benefits’ and ‘harms’). The disagreement is about how these ‘benefits’ or ‘harms’ should be understood, whose benefits should be taken into account and how much benefit NBS should produce.^25^

In the following, we will distinguish different ways in which NBS influences lives by using the terms ‘hard’ and ‘soft’ impacts from philosophy of technology. ‘Hard’ impacts are typically quantifiable in terms of number of affected children or life years gained and constitute the most broadly accepted focus of NBS; ‘soft’ impacts refer to psychosocial impacts that influence the experienced quality of life and need to be described in qualitative terms.^26^ The question whether and to what extent NBS is allowed to produce soft impacts is much more controversial, as this concerns people's private lives and does not fit within the presupposed liberal constraints.

##### Hard impacts

Health is a ‘hard impact’ of NBS and is the least controversial value in the ethical debate on NBS. Wilson and Jungner's standard for screening programs (1968), which still shapes the debate about NBS panels, stated that screening is justified if it offers a significant health benefit that clearly outweighs the risks and side effects of screening. Some authors argue strictly according to the Wilson and Jungner criteria and claim that we ought only to disclose NBS results that produce a direct and immediate health benefit for children.^27^ They thus impose limitations on the intrusion that NBS is allowed to have in the lives of people. Others add that extending the focus of NBS beyond health would make NBS more controversial, as parents may not want to receive information about untreatable diseases, risk information, late‐onset diseases or carrier status. Consequently they may refuse to participate in NBS, meaning children with treatable diseases might go undetected.^28^

While ‘health’ is the original and uncontroversial focus of NBS, there has perhaps always been disagreement as to how ‘treatable’ a disease needs to be to be included in NBS.^29^ Some authors, such as Bailey and Wolf, argue that NBS should also return information about untreatable diseases as it enables early entry into intervention services that contribute to improvement of a child's condition.^30^ Others argue that intervention services do not always improve the health of children, as physicians do not always have the required knowledge about rare diseases, which influences the quality of their decisions about what intervention should be administered, whether to restrict a child's access to costly treatment, or start palliative treatment.^31^ Even if some children with untreatable rare diseases are well attended to, we should not assume this will happen for all children.

Others take a broader perspective on the benefits of the inclusion: early detection of children with these diseases allows for the studying of incidence, symptoms and effects of early intervention, and enables inclusion of a child in clinical trials. Therefore screening for untreatable diseases may yield health benefits for the child, as well as the entire future population of patients with the same disease.^32^ Paul, however, criticizes the promise that research will benefit the child's health, pointing out that this line of thought mistakes research for treatment, and that no health benefit should be expected from research.^33^

NBS can also disclose information not immediately relevant for the infant's health, such as information about carrier status, late‐onset diseases or risks. Carrier status information, for example, will only become relevant for choices the child makes about his or her own reproduction. For some authors this irrelevance to the child's health is sufficient reason to reject its disclosure after NBS.^34^ Others argue carrier status information is of more immediate importance as it can inform parents about their own carrier status and their risk of having affected future children, and can allow them to make informed decisions about their further reproduction.^35^ It is observed, however, that parents often do not understand or use carrier status information or misinform their own children when they grow up.^36^ In addition, some authors argue against the inclusion of carrier status information in NBS on the grounds that NBS should not be used as a surrogate for carrier screening of parents for this exceeds the goal of NBS.^37^ If parents want this information, they can ask a doctor and do not need NBS.^38^

Arguments in favor of the return of risk information or late(r) onset diseases after NBS state that it helps to be more attentive to signs of disease, permitting swift action to prevent harm.^39^ It enables parents, for example, to see symptoms of inherited diabetes 1 in time and avoid that their child will be diagnosed in an emergency setting, or it helps physicians to recognize symptoms of rare diseases they are unfamiliar with.^40^ However, given the psychosocial harms of knowledge about risk and late‐onset diseases, described below, authors such as Ross advise against disclosure if there is no prevention strategy.^41^

##### Soft impacts

Soft impacts are hotly debated and refer to outcomes of NBS that influence human socio‐emotional wellbeing, such as psychosocial harms or benefits that families may experience when they receive information about risks, late(r)‐onset diseases, or carrier status information. Benefits include a sense of relief to know that a particular child did not inherit a family disease, or empowerment of parents to make their own decisions about their future reproduction when they receive this information.^42^ Disclosure of information about late(r)‐onset or untreatable diseases also helps avoiding the so‐called ‘diagnostic odyssey’; a long and burdensome search for a diagnosis, which children with rare diseases often undergo because physicians have difficulty recognizing the symptoms and requesting the appropriate diagnostic texts.^43^ Having a diagnosis of untreatable diseases has intrinsic importance, according to Botkin and Rothwell, who refer to empirical studies that suggest it helps parents to move toward acceptance of the disease and organize their lives around it.^44^ Others are more critical of this soft benefit, arguing that shortening the diagnostic odyssey should not be the task of NBS; it can also be shortened if pediatricians’ ability to recognize these diseases is improved.^45^

Many authors also expect soft harms from the disclosure of untreatable diseases, late(r)‐onset diseases, risk information and carrier status, as their disclosure can produce parental feelings of anxiety, stress and guilt, resulting in disrupted parent‐child bonding, overprotection, damage to the child's self‐esteem, and diminished wellbeing. Expected social harms include stigmatization or discrimination within the family or in society, which may lead to difficulties to form relationships later in life, make reproductive decisions, obtain insurance, buy a house, or find a job.^46^

An issue particular to adult‐onset diseases or risk information is medicalization of the healthy life of a child, or the creation of ‘patients in waiting’: people diagnosed with a disorder and ‘waiting’ for its symptoms to develop.^47^ As children ‘at risk’ may be treated differently by parents, they can develop disordered illness behavior later in life such as excessive worries about health.^48^ The uncertain nature of risk information may cause ‘(…) a type of social identity in which people are neither well nor ill, but “at risk”.’^49^ This knowledge might interfere with life plans, as it may medicalize nonmedical decisions about marriage, children, and career.^50^

This debate reveals that NBS influences aspects of people's private lives, and raises the question which soft impacts NBS should produce (if any). Interestingly, some authors claim that NBS policymakers should not answer this question. As little is known about the actual occurrence of soft impacts, how parents evaluate them and cope with them, they argue parents themselves should determine the value of this information.^51^ Botkin and Rothwell suggest, for example, to adopt a notion of ‘personal utility’, which refers to the use parents can make of disease information in their own lives.^52^ They argue that parents should make their own private decision about the kind of disease information they want to obtain.

The term ‘personal benefit’ suggests that NBS offers information and individuals develop their own understanding of its benefits in their private lives. Some authors, however, speak of ‘family benefits’ since many soft impacts are difficult to individualize, such as the presumed benefit of an early diagnosis to help parents create a well‐organized, stable, and caring environment for the child, benefitting the whole family.^53^ Authors such as Forman and colleagues choose social terms to refer to these benefits; such as ‘family benefits’ because they think NBS helps families foster a caring relationship.^54^ Rare authors, such as Ross and Knoppers, speak about ‘relational duties’ arising from the shared nature of genes, urging parents to warn blood relatives who may be affected,^55^ or puzzling healthcare providers confronted with parents who selectively inform their family members.^56^

Characterizing soft impacts as personal or family values allows to appreciate NBS’ influence on people's private lives and raise the question how this influence ought to be shaped and limited. Most authors however say that parents should decide about soft impacts privately. Considerations about parental duties are rare, as well as calls to involve a broad variety of stakeholders—including clinicians, researchers, ethicists, public health professionals, policymakers, and patients—in thinking about what/whose benefits should be the focus of NBS^57^ or that the public of parents at large should be involved in establishing the goals of NBS policy.^58^

#### 3.1.4 Dealing responsibly with results that are not included in the program

Some ways in which NBS influences people's lives are beyond the control of NBS policymakers. A recurring theme in the literature is how to deal with results NBS does not aim for, such as false positives, so‐called incidental, unsolicited or unanticipated findings, and uncertain or ambiguous test results and associated problems of overdiagnosis or underdiagnosis (see Table 2). Unexpected findings have been part of NBS since the beginning, but attract more attention at the prospect of introducing NGS in NBS, as this may lead to an increase in the number and variety of results not included in the program.

Insofar as these findings reveal untreatable variants of diseases, late(r)‐onset diseases, risk information or carrier status, the issues addressed in the literature largely mimic the hard and soft benefits and harms brought forward in the previous section.^59^ Uncertain findings, however, raise a slightly different set of problems, as a lack of knowledge about prognosis or effects of treatment makes adequate counseling difficult.^60^ NBS may produce all kinds of information of unknown clinical significance as a by‐product, especially once NGS is introduced.^61^ Such findings do not clearly indicate that a child is diseased, nor that the child is healthy, thus complicating treatment decisions.^62^ Treating the child as diseased may lead to harmful overtreatment, it can produce aforementioned psychosocial effects and adversely impact access to life‐saving and costly medical interventions, such as heart or lung transplants.^63^ But treating children as healthy comes with the risk of the child developing symptoms which early treatment could probably have prevented.^64^

Additionally, some authors claim that results that are not included in the program may negatively impact parents’ trust in NBS, as they do not anticipate these findings and may not want them.^65^ Consequently they may refuse to subject their next child to screening, which would make NBS less effective in detecting affected children. Trust in NBS is therefore considered fragile in view of results that do not fit the NBS program, as they influence the private lives of people inadvertently.

### 3.2 The manner in which the public is involved

It is typical for liberal perspectives that policymakers should refrain from influencing people's decisions about how they want to lead their private lives. This individual freedom is, however, restricted when the choices people make harm themselves or others. The debate about whether NBS ought to be mandatory or voluntary revolves around the question whether refusal of NBS justifies government intervention in personal choice. The main argument supporting mandatory screening is that society's obligation to promote a child's health supersedes parental prerogatives to choose and possibly refuse NBS.^66^ This consequently sets ‘high standards’ for the program, as making NBS mandatory is only justified if it promotes the health of the infant.^67^ Adding diseases whose early detection does not satisfy this requirement, as well as the possibility of findings that are not targeted by the program, thus immediately raise the question whether screening should become subject to consent.^68^ Information that does not immediately serve the health of a child should not be provided in mandatory NBS, according to some authors, as people may disagree about its desirability. In these cases, mandatory screening is not justified, and informed consent should be asked.^69^

The most important arguments in favor of voluntary and informed participation in NBS include that NBS should respect autonomy, and that parents should have the responsibility for the healthcare decisions for their children.^70^ Voluntary screening requires informing parents and asking their consent, which is expected to prepare parents for screening and to lead to their more active participation in NBS. This would motivate them to promptly take action in the case of a positive result after NBS, improving the effectiveness of screening.^71^ Some authors even state that informed consent enhances trust in the healthcare system, for it fosters the idea of ‘partnership’ between parents and professionals in providing care for the child.^72^

While arguments for mandatory and voluntary screening are in direct opposition, mandatory, and voluntary programs often do not differ much in practice. In mandatory NBS, parents are informed prior to screening and are allowed to opt out on grounds of personal or religious reasons.^73^ And the uptake of voluntary and mandatory programs is equally high, causing some authors to argue that mandatory NBS is unnecessary if maximum uptake is intended.^74^ The most pressing discussions in this context concern whether and how the arguments that traditionally supported voluntary or mandatory screening can continue to justify NBS when future screening technologies might disclose more disease information or results that are not included in the program.

#### 3.2.1 Difficulties related to mandatory programs

In contexts where NBS is mandatory, nowadays there is more support to make NBS at least partly voluntary. As expanding NBS programs yield more and different types of information, many authors believe that results exceeding the original conception of benefit (health) will influence so‐called ‘private’ values, such as values concerning beliefs about child bearing, child rearing, family, and future reproductive decisions. They think parents should be allowed to make choices based on these values in their private lives and be given the opportunity to give or refuse their consent.^75^

Several authors have argued for new models of participation, which make one part of the program mandatory, while making another part with no immediate benefit for the child optional.^76^ Tarini and Goldenberg support this idea, but remark that ‘(…) parents may misunderstand the difference between mandatory and voluntary screening, and then may choose to forego screening for mandatory conditions’.^77^ This can happen, because mandatory screening can still be refused, for example, on religious grounds. Ross is less afraid of this risk and instead proposes adopting an opt‐out procedure for treatable diseases, allowing parents the possibility to choose while also nudging them toward consent, and an opt‐in or more formal consent process for other conditions.^78^ The advantage of such an approach would be that information is needed for both tiers and parental autonomy is respected, but in tier two more information needs to be offered to support parental consent. Disadvantages are that new knowledge about diseases included in NBS necessitates a continuous revision of the two tiers. Furthermore, screening for tier one diseases may occasionally identify diseases belonging to tier two, and healthcare professionals need to be well‐educated about NBS to explain to parents the difference between both tiers.^79^

#### 3.2.2 Difficulties in voluntary programs

In voluntary programs, expansions of the NBS panel increases the complexity and volume of information that needs to be provided to parents.^80^ Informing is difficult for it needs to be done in a limited time, yet should be sufficient, and avoid being alarmist about diseases that will occur very rarely.^81^ Information should be given when parents are receptive to it, by knowledgeable healthcare providers who are able to attune information to the socio‐cultural context of parents.^82^ However, many authors warn that healthcare professionals may not inform parents well when NBS expands, as they lack knowledge about the rare diseases included in NBS, and the results that are not aimed for by the program, making it difficult to enable parents to give truly informed consent.^83^ Parents, on the other hand, may be unable to grasp the information provided and their eventual consent may be perfunctory.^84^ The introduction of genetic technologies will only increase these perceived problems of giving and receiving information.^85^

To avoid an information overload for parents, authors discuss the type of informed consent procedure that should be adopted. Formal informed consent procedures require giving extensive information and asking for a signature from parents; more informal informed consent procedures allow informing parents in more general terms and giving them the chance to opt‐out.^86^ Some argue that generic consent suffices when NBS serves the health of babies,^87^ others suggest parents should be offered the possibility to choose from a more differentiated ‘menu of options’ about which they need to be informed more extensively.^88^ What model of informed consent is adopted depends, however, on the purpose of the program and its expected acceptance by society as well as on the potential findings that are not aimed for by the program. Some authors fear that these findings are detrimental to parental trust in NBS.^89^ Others focusing on this problem of ‘incidental’ or ‘unsolicited’ findings, suggest that trust can be preserved if consent is asked for the disclosure of these findings too.^90^ Authors opposing this idea argue that NBS should serve the infant's health; especially disclosure of incidental findings that are relevant to other family members is problematic as it infringes the right not to know of these family members.^91^ Another group of authors suggests making a distinction between different kinds of information: they say, for example, that *only* incidental genetic variants which point to serious and actionable health problems should be disclosed.^92^

In all these reflections, however, what is negotiated is how the personal and private choice of parents about their use of NBS for the care of their children ought to be fostered by means of information, even if NBS offers them an abundance of (sometimes unexpected) results.

## 4 CONCLUSION AND DISCUSSION

Our analysis of the literature yields an overview of the issues and values that play a role in the discussion, which we presented in Table 3. The most striking result of our analysis is that anticipated expansions of the NBS program beyond its original focus on health improvement for children, lead to more calls to make participation in NBS (at least partially) voluntary. As mandatory screening rests on the argument that the state may act paternalistically if that would protect the health of babies, the support for mandatory screening diminishes when expansions of NBS impact values that concern not only the protection of health, but also choices that people make in their private lives.

This connection between the justification of the NBS program and the growing support for voluntary NBS, is in our view indicative of the liberal presuppositions figuring in the background of the ethical debate about NBS. Liberals generally separate between issues that belong to the public domain and should be decided on by policymakers, and private issues that are the object of informed consent.^93^ Naturally, liberals also disagree about where to draw the distinction between public and private realms, allowing for narrower and broader understandings of the responsibilities of policymakers. Its narrowest conception only allows the state to exercise power over individual citizens if ‘it is to prevent harm to others’.^94^ In line with this approach, some authors argue that state power to mandate NBS is justified if this protects the health of children, but they oppose mandated NBS when it influences not just health, but other aspects of private life.

Relegating some issues to the private domain, however, may also function as an effective discussion stopper. Hard impacts such as health are considered the responsibility of policymakers in the public domain. These impacts on health are backed up by scientific evidence that provides quantified justification for the benefits of NBS. While there is disagreement about the convincingness of this evidence, focusing the ethics of NBS on impacts on health at least reduces these disagreements and makes them more manageable as including the disease in NBS will enable more research and will allow providing more evidence in the future.^95^ Soft impacts, by contrast, invite a lot of disagreement that seems a lot more challenging to manage; as the literature shows, authors disagree as to whether they occur, how they should be evaluated, and by whom. More research will be unlikely to settle these disagreements as it will only allow one to acquire an empirical overview over the values that parents and professionals generally hold dear, but research remains silent about what values an NBS program *ought* to pursue or foster. Assigning responsibility for soft impacts to the private domain, removes these controversial topics from the public arena and burdens parents with them.

This division of ethical labor mimics what happens in many other ethical debates, about new technologies for society; hard impacts are often considered worthy of policy concern, while concern for soft ones is delegated to the private domain.^96^ The downside of this division of ethical labor is, however, that parents are charged with quite a difficult task for which they receive little support. The NBS literature shows that it is hard to provide the necessary information to help parents make an informed decision. Professional ethical reflection does not help them to decide either: ethicists do name the issues that may occur in people's private lives, but restrict their ethical reflection and argumentation to the policy decisions. Parents therefore have to make their choices without the support of ethicists who are trained in ethical reflection and who could therefore be expected to enhance their reflection and give it more substance.

In our view, soft impacts caused by NBS deserve more attention in ethical reflection, as they offer possibilities whose value for the care of children should be taken into account. One of the ways to achieve this is by means of fostering a dialogue in which parents of healthy and diseased children share their experience when they obtain different kinds of health information and explain how it is valuable, or not. This allows them to broaden the limited scope of their own experience and enhance their reflection on what good care for their children consists in and how NBS can and cannot contribute to it.

Such a dialogue, furthermore, should be coupled with the development of a more substantive evaluative language which exceeds risks, benefits, and harms that dominate ethical reflections about NBS. Our review allowed us to encounter such substantive terms in the work of authors who tentatively use a relational vocabulary (‘family benefits’, ‘caring bonds’, ‘trust’, ‘solidarity with future patients’) to convey a message about what may be gained if NBS is allowed to give information that can be used to strengthen and foster caring relationships—such as when information about untreatable diseases allows parents to respond to the needs of the child, organize professional care, and seek a new equilibrium in their family (including other siblings) as well as their jobs and extended social network which suits everyone's lives, joys, and ambitions.

While terms such as ‘social benefits’, ‘care’, ‘trust’, and ‘solidarity’ suggest a communitarian or care ethics perspective on NBS, it has never been developed further to supplement the predominant liberal perspective on NBS. Some authors such as Françoise Baylis and colleagues have suggested developing a feminist relational approach to public health ethics, but focused primarily on vaccination programs as an example. Using notions such as solidarity, reciprocity, mutual dependency, and relational autonomy, they argue that individuals depend on each other to become and stay healthy and autonomous decision‐makers. Public health programs such as vaccination would, in their view, be difficult to defend without a proper grasp of how people are related: the meaning of ‘public interest’ and ‘common good’, which ought to be the focus of public health programs, requires understanding how citizens depend on each other and need each other.^97^

This solidarity approach by Baylis et al. inspired our own proposal for the future development of ethical debate about NBS. While we realize that the health of the population does not depend in the same way on the solidarity of individuals in NBS as it does in a vaccination program, we do think that it makes sense in the context of NBS too. The dependency of newborn children on their parents’ care, the blood relatedness of family members and disease inheritance within a family, as well as connectedness of a patient to distant (future) others with the same (rare) disease, provide a lead to rethink what the common good is and what solidarity would accordingly require in a context where technologies are able to provide an abundance of information about the health of an infant. It would go beyond the scope of this article to actually elaborate such a social perspective to the common good in NBS, but we listed the questions that we think are a valuable starting point for its development in Table 4. These questions invite empirical research and theoretical reflection on the meaning and normative content of concepts such as care, trust, and solidarity in the context of an expanding NBS program, as well as on whether and how NBS policy can continue to respect the distinction between the public and private domain. This work would enhance the debate about NBS, which is nowadays hampered by the liberal choice to delegate the responsibility for very difficult issues to parents in the private domain.

## CONFLICT OF INTEREST

The authors declare no conflict of interest.

## Footnotes

1
In our article we focus on blood‐based NBS of noninfectious disorders. We exclude all other types of screening performed on newborns, such as a hearing test or pulse oximetry.

2
Lindee, M. S. (2005). *Moments of truth in genetic medicine* Baltimore, MD: JHU Press; Paul, D. B., & Brosco, J. P. (2013). *The PKU paradox: A short history of a genetic disease*. Baltimore, MD: JHU Press.

3
Burgard, P., Cornel, M., Di Filippo, F., Haege, G., Hoffmann, G. F., Lindner, M., … Vittozzi, L. (2012). *Report on the practices of newborn screening for rare disorders implemented in member states of the European Union, candidate, potential candidate and EFTA countries* Luxembourg: European Commission; Borrajo, G. J. (2007). Newborn screening in Latin America at the beginning of the 21st century. *Journal of Inherited Metabolic Disease*, *30*, 466–481; Therrell, B. L., & Adams, J. (2007). Newborn screening in North America. *Journal of Inherited Metabolic Disease*, *30*, 447–465.

4
Service, R. F. (2006). Gene sequencing. The race for the $1000 genome. *Science (New York, NY), 311*, 1544–1546; Levy, S., Sutton, G., Ng, P. C., Feuk, L., Halpern, A. L., Walenz, B. P., … Venter, J. C. (2007). The diploid genome sequence of an individual human. *PLoS Biology*, *5*, e254. 10.1371/journal.pbio.0050254PMC196477917803354

5
See for example: Pettit, P. (1994). Liberal/Communitarian: MacIntyre's mesmeric dichotomy. In J. Horton & S. Mendus (Eds.), *After MacIntyre; Critical perspectives on the work of Alasdair MacIntyre* (pp. 176–204). Notre Dame, IN: University of Notre Dame Press and Stout, J. (2004). *Democracy and tradition*. New Jersey, NJ: Princeton University Press.

6
In addition to these two themes, issues related to the justification of storage, governance, ownership and use of residual bloodspots after NBS also feature in the ethical literature on NBS. While relationships play a role here too, we chose to exclude this theme here because it is not exclusively discussed in the ethical literature about NBS, since residual blood may also be retained and stored after regular diagnoses or research and a presentation of the ethical issues raised about storage in the NBS literature would therefore be incomplete.

7
Baily, M. A., & Murray, T. H. (2008). Ethics, evidence, and cost in newborn screening. *Hastings Center Report*, *38*, 23–31; Elliman, D. (2012). Ethical aspects of the expansion of neonatal screening programme due to technological advances. *Clinical Chemistry and Laboratory Medicine*, *50*, 999–1002; Dhondt, J. L. (2010). Expanded newborn screening: Social and ethical issues. *Journal of Inherited Metabolic Disease*, *33*, S211–S217; de Montalembert, M., Bonnet, D., Lena‐Russo, D., & Briard, M. L. (2005). Ethical aspects of neonatal screening for sickle cell disease in Western European countries. *Acta Paediatrica*, *94*, 528–530; Farrell, P. M. (2008). Is newborn screening for cystic fibrosis a basic human right? *Journal of Cystic Fibrosis*, *7*, 262–265; Autti‐Ramo, I., Mäkelä, M., Sintonen, H., Koskinen, H., Laajalahti, L., Halila, R., … Tyni, T. (2005). Expanding screening for rare metabolic disease in the newborn: An analysis of costs, effect and ethical consequences for decision‐making in Finland. *Acta Paediatrica*, *94*, 1126–1136; Avard, D., Vallance, H., Greenberg, C., & Potter, B. (2007). Newborn screening by tandem mass spectrometry ‐ Ethical and social issues. *Canadian Journal of Public Health*, *98*, 284–286; Bailey, D. B., Skinner, D., Davis, A., Whitmarsh, I., & Powell, C. (2008). Ethical, legal, and social concerns about expanded newborn screening: Fragile X syndrome as a prototype for emerging issues. *Pediatrics*, *121*, E693–E704; Forman, J., Coyle, F., Levy‐Fisch, J., Roberts, P., Terry, S., & Legge, M. (2013). Screening criteria: The need to deal with new developments and ethical issues in newborn metabolic screening. *Journal of Community Genetics*, *4*, 59–67; Levy, P. A. (2010). An overview of newborn screening. *Journal of Developmental and Behavioral Pediatrics*, *31*, 622–631; Matsuda, I. (2003). Bioethical considerations in neonatal screening: Japanese experiences. *Southeast Asian Journal of Tropical Medicine and Public Health*, *34*(Suppl 3), 46–48; Saxena, A. (2003). Issues in newborn screening. *Genetic Testing*, *7*, 131–134; Wilcken, B. (2003). Ethical issues in newborn screening and the impact of new technologies. *European Journal of Pediatrics*, *162*, S62–S66; Wilfond, B., & Rothenberg, L. S. (2002). Ethical issues in cystic fibrosis newborn screening: From data to public health policy. *Current Opinion in Pulmonary Medicine*, *8*, 529–534; Knoppers, B. M., Sénécal, K., Borry, P., & Avard, D. (2014). Whole‐genome sequencing in newborn screening programs. *Science Translational Medicine*, *6*, 229cm2; Howard, H. C., Knoppers, B. M., Cornel, M. C., Wright Clayton, E., Sénécal, K., & Borry, P. (2015). Whole‐genome sequencing in newborn screening? A statement on the continued importance of targeted approaches in newborn screening programmes. *European Journal of Human Genetics*, *23*, 1593–1600; Roberts, J. S., Dolinoy, D., & Tarini, B. (2014). Emerging issues in public health genomics. *Annual Review of Genomics and Human Genetics*, *15*, 461–480; Leao, L. L., & Aguiar, M. J. (2008). Newborn screening: What pediatricians should know. *Jornal de Pediatria (Rio J)*, *84*, S80–S90; Spahis, J. K., & Bowers, N. R. (2006). Navigating the maze of newborn screening. *MCN. The American Journal of Maternal Child Nursing*, *31*, 190–196. Green, N. (2009). Every child is priceless: Debating effective newborn screening policy. *Hastings Center Report*, *39*, 6–7; author reply: 7–8.

8
Spahis and Bowers, *op. cit*. note 7.

9
Golden‐Grant, K., Merritt, J. L. II, & Scott, C. R. (2015). Ethical considerations of population screening for late‐onset genetic disease. *Clinical Genetics*, *88*, 589–592; Mak, C. M., Lee, H. C., Chan, A. Y., & Lam, C. W. (2013). Inborn errors of metabolism and expanded newborn screening: Review and update. *Critical Reviews in Clinical Laboratory Sciences*, *50*, 142–162. 10.1111/cge.1256625677830

10
Roberts et al., *op. cit*. note 7.

11
Leao and Aguiar, *op. cit* note 7; Spahis and Bowers, *op. cit*. note 7.

12
Roberts et al., *op. cit*. note 7.

13
Knoppers et al., *op. cit* note 7; Howard et al., *op. cit*. note 7.

14
Baily and Murray, *op. cit* note 7; Forman et al., *op. cit* note 7; Miller, F. A. (2009). The complex promise of newborn screening. *Indian Journal of Medical Ethics*, *6*, 142–148; Paul, D. B. (2008). Patient advocacy in newborn screening: Continuities and discontinuities. *American Journal of Medical Genetics*, *148C*, 8–14; Moyer, V. A., Calonge, N., Teutsch, S. M., Botkin, J. R.; United States Preventive Services Task Force. (2008). Expanding newborn screening: Process, policy, and priorities. *Hastings Center Report*, *38*, 32–39. 10.1353/hcr.0.001118581935

15
Miller, *op. cit*. note 14.

16
Moyer et al., *op. cit*. note 14.

17
Wilcken, *op. cit* note 7; Bailey, D. B. Jr., Skinner, D., & Warren, S. F. (2005). Newborn screening for developmental disabilities: Reframing presumptive benefit. *American Journal of Public Health*, *95*, 1889–1893; Wilcken, B. (2007). Recent advances in newborn screening. *Journal of Inherited Metabolic Disease*, *30*, 129–133. Historical studies support this criticism of the scientific justification for NBS. They describe that little was known even about PKU when national PKU screening programs were initially launched; mildly and more seriously affected children were treated in the same way, thus producing cognitive deficits in patients who were overtreated (Paul, *op. cit* note 14; Mitchell, M. X. (2017). Screening out controversy: Human genetics, emerging techniques of diagnosis, and the origins of the social issues Committee of the American Society of Human Genetics, 1964–1973. *Journal of the History of Biology*, *50*, 425–456). Authors also sometimes shortly comment that if sufficient evidence should be provided that points out how the costs and benefits of NBS should be balanced to all alternative ways to spend money on health care for children, we would never have preventive programs as providing this evidence would be too complex and time‐consuming. (Howell, R. R. (2009). Every child is priceless: Debating effective newborn screening policy. *Hastings Center Report*, *39*, 4–6).

18
Golden‐Grant et al., *op. cit* note 9; Bunnik, E. M., de Jong, A., Nijsingh, N., & de Wert, G. M. (2013). The new genetics and informed consent: Differentiating choice to preserve autonomy. *Bioethics*, *27*, 348–355; Dondorp, W. J., de Wert, G. M., & Niermeijer, M. F. (2012). Genomic sequencing in newborn screening programs. *JAMA*, *307*, 2146; author reply: 2147; Ciarleglio, L. J., Bennett, R. L., Williamson, J., Mandell, J. B., & Marks, J. H. (2003). Genetic counseling throughout the life cycle. *Journal of Clinical Investigation*, *112*, 1280–1286; Scala, I., Parenti, G., & Andria, G. (2012). Universal screening for inherited metabolic diseases in the neonate (and the fetus). *Journal of Maternal‐Fetal and Neonatal Medicine*, *25*, 4–6; Kerruish, N. J., & Robertson, S. P. (2005). Newborn screening: New developments, new dilemmas. *Journal of Medical Ethics*, *31*, 393–398; Berg, J. S., & Powell, C. M. (2015). Potential uses and inherent challenges of using genome‐scale sequencing to augment current newborn screening. *Cold Spring Harbor Perspectives in Medicine*, *5*, a023150; Stewart, F. (2006). Ethics of newborn screening. *Current Pediatrics*, *16*, 216–220; Almond, B. (2006). Genetic profiling of newborns: Ethical and social issues. *Nature Reviews Genetics*, *7*, 67–71; Reinstein, E. (2015). Challenges of using next generation sequencing in newborn screening. *Genetics Research*, *97*, e21. Phadke, S., & Gowda, M. (2013). Genetic testing in children. *Indian Pediatrics*, *50*, 823–827. 10.1111/bioe.1203023718722

19
Knoppers et al., *op. cit* note 7; Abbing, H. D. (2004). Neonatal screening, new technologies, old and new legal concerns. *European Journal of Health Law*, *11*, 129–137. 10.1163/157180904145743115387421

20
Golden‐Grant et al., *op. cit* note 9; Almond, *op. cit* note 19; Reinstein, *op. cit*. note 19.

21
Roberts et al., *op. cit*. note 7.

22
Wilcken *op. cit* note 7; Miller, *op. cit* note 14; Berg and Powell, *op. cit* note 19; Stewart, *op. cit* note 19; Khoury, M. J., McCabe, L. L, & McCabe, E. R. (2003). Population screening in the age of genomic medicine. *New England Journal of Medicine, 348*, 50–58; Walters, L. (2012). Genetics and bioethics: How our thinking has changed since 1969. *Theoretical Medicine and Bioethics, 33*, 83–95; Committee on Bioethics. (2001). Ethical issues with genetic testing in pediatrics. *Pediatrics*, *107*, 1451–1455; Grosse, S. D., Rogowski, W. H., Ross, L. F., Cornel, M. C., Dondorp, W. J., & Khoury, M. J. (2010). Population screening for genetic disorders in the 21st century: Evidence, economics, and ethics. *Public Health Genomics*, *13*, 106–115; McCabe, L. L., & McCabe, E. R. (2004). Genetic screening: Carriers and affected individuals. *Annual Review of Genomics and Human Genetics, 5*, 57–69; Cornel, M. C., Gille, J. J., Loeber, J. G., Vernooij‐van Langen, A. M., Dankert‐Roelse, J., & Bolhuis, P. A. (2012). Improving test properties for neonatal cystic fibrosis screening in the netherlands before the nationwide start by May 1st 2011. *Journal of Inherited Metabolic Disease, 35*, 635–640. 10.1007/s10545-012-9452-7PMC338825122302635

23

Ross, L. F. (2012). Newborn screening for sickle cell disease: Whose reproductive benefit? European Journal of Human Genetics, 20, 484–485. 2207189210.1038/ejhg.2011.191PMC3330207

24
Paul, *op. cit* note 14; Miller, F. A., Robert, J. S., Hayeems, R. Z. (2009). Questioning the consensus: Managing carrier status results generated by newborn screening. *American Journal of Public Health*, *99*, 210–215. 10.2105/AJPH.2008.136614PMC262276819059852

25
Forman et al., *op. cit* note 7; Wilcken, *op. cit* note 7; Knoppers et al., *op. cit* note 7; Bailey et al., *op. cit* note 17; Wilcken, *op. cit* note 17; Berg and Powell, *op. cit* note 19; Botkin, J. R., & Rothwell, E. (2016). Whole genome sequencing and newborn screening. *Current Genetic Medicine Reports*, *4*, 1–6; Pollitt, R. J. (2010). New technologies extend the scope of newborn blood‐spot screening, but old problems remain unresolved. *Acta Paediatrica*, *99*, 1766–1772; Wieser, B. (2010). Public accountability of newborn screening: Collective knowing and deciding. *Social Science and Medicine*, *70*, 926–933; Wilcken, B., & Wiley, V. (2015). Fifty years of newborn screening. *Journal of Paediatrics and Child Health*, *51*, 103–107; Arnold, C. G. (2014). Two faces of patient advocacy: The current controversy in newborn screening. *Journal of Medical Ethics*, *40*, 558–562; O'Leary, P., & Maxwell, S. (2015). Newborn bloodspot screening policy framework for Australia. *Australasian Medical Journal*, *8*, 292–298; Tarini and Goldenberg, *op. cit* note 7; Trotter, T. L., Fleischman, A. R., Howell, R. R., Lloyd‐Puryear, M.; Secretary's Advisory Committee on Heritable Disorders in Newborns and Children. (2011). Secretary's Advisory Committee on Heritable Disorders in Newborns and Children response to the President's Council on Bioethics report: The changing moral focus of newborn screening. *Genetics in Medicine*, *13*, 301–304; Milliez, J. (2009). Ethical aspects concerning neonatal screening FIGO Committee for the Ethical Aspects of Human Reproduction and Women's Health. *International Journal of Gynecology and Obstetrics*, *106*, 273–274; Clayton, E. W. (2010). Currents in contemporary ethics. State run newborn screening in the genomic era, or how to avoid drowning when drinking from a fire hose. *Journal of Law, Medicine and Ethics*, *38*, 697–700. 10.1111/j.1748-720X.2010.00522.x20880251

26
Swierstra, T., & te Molder, H. (2012). Risk and soft impacts. In S. Roeser, R. Hillerbrand, P. Sandin, & M. Peterson (Eds.), *Handbook of risk theory* (pp. 1049–1066). Dordrecht, the Netherlands: Springer.

27
Abbing, *op. cit* note 20; Clayton, E. W. (2003). Ethical, legal, and social implications of genomic medicine. *New England Journal of Medicine*, *349*, 562–569; Ross, L. F. (2012). Newborn screening for lysosomal storage diseases: An ethical and policy analysis. *Journal of Inherited Metabolic Disease*, *35*, 627–634. 10.1007/s10545-011-9435-022189599

28
Berg and Powell, *op. cit* note 19; Baily, M. A., Becker, W. Jr, Hayes, M., Clayton, E. W., Grosse, S. (2005). Exploring options for expanded newborn screening. *Journal of Law, Medicine and Ethics, 33*, 46–48. 16689158

29
Paul, *op. cit*. note 14.

30
Bailey et al., *op. cit* note 17; Wolf, B. (2015). Why screen newborns for profound and partial biotinidase deficiency? *Molecular Genetics and Metabolism*, *114*, 382–387. 10.1016/j.ymgme.2015.01.00325638506

31

Deem, M. J. (2016). Whole‐genome sequencing and disability in the NICU: Exploring practical and ethical challenges. Pediatrics, 137(Suppl 1), S47–S55. 2672970310.1542/peds.2015-3731IPMC9923973

32
Bailey et al., *op. cit* note 17; Khoury et al., *op. cit* note 23; Trotter et al., *op. cit* note 26; Wolf, *op. cit* note 31; Mitchell, *op. cit*. note 18.

33
Paul, *op. cit*. note 14.

34
Elliman, *op. cit*. note 7.

35
Bailey et al., *op. cit* note 17; Bombard, Y., & Miller, F. A. (2012). Reply to Ross' commentary: Reproductive benefit through newborn screening: Preferences, policy and ethics. *European Journal of Human Genetics*, *20*, 486–487. 10.1038/ejhg.2012.25PMC333023222378289

36
Miller et al., *op. cit* note 25; Noke, M., Wearden, A., Peters, S., & Ulph, F. (2014). Disparities in current and future childhood and newborn carrier identification. *Journal of Genetic Counseling*, *23*, 701–707; DeLuca, J., Zanni, K. L., Bonhomme, N., & Kemper, A. R. (2013). Implications of newborn screening for nurses. *Journal of Nursing Scholarship*, *45*, 25–33. 10.1007/s10897-014-9740-525009079

37
Roberts et al., *op. cit* note 7; Committee on Bioethics, *op. cit* note 23; Noke et al., *op. cit*. note 37.

38
Miller et al., *op. cit*. note 25.

39
Berg and Powell, *op. cit* note 19; Committee on Bioethics, *op. cit* note 23; Wolf, *op. cit*. note 31.

40
Wolf, *op. cit* note 31; Ross, L. F. (2007). Against newborn screening for type 1 diabetes. *Archives of Pediatrics and Adolescent Medicine*, *161*, 616–617; author reply: 617–618. 10.1001/archpedi.161.6.616-c17548771

41
Ross, L. F. (2003). Minimizing risks ‐ The ethics of predictive diabetes mellitus screening research in newborns. *Archives of Pediatrics and Adolescent Medicine*, *157*, 89–95; Ross, *op. cit*. note 41. 10.1001/archpedi.157.1.8912517201

42
Committee on Bioethics, *op. cit* note 23; Bombard and Miller, *op. cit* note 36; Fletcher, J. M. (2006). Screening for lysosomal storage disorders–A clinical perspective. *Journal of Inherited Metabolic Disease*, *29*, 405–408. 10.1007/s10545-006-0246-716763909

43
Wilcken, *op. cit* note 7; Miller, *op. cit* note 14; Bailey et al., *op. cit* note 17; Ciarleglio et al., *op. cit* note 19; Berg and Powell, *op. cit* note 19; Khoury et al., *op. cit* note 23; Walters, *op. cit* note 23; Fletcher, *op. cit*. note 43.

44
Botkin and Rothwell, *op. cit*. note 26.

45

Ross, L. F. (2006). Screening for conditions that do not meet the Wilson and Jungner criteria: The case of Duchenne muscular dystrophy. American Journal of Medical Genetics, 140A, 914–922. 10.1002/ajmg.a.3116516528755

46
Elliman, *op. cit* note 7; Dhondt, *op. cit* note 7; Bailey et al., *op. cit* note 7; Leao and Aguiar, *op. cit* note 7; Golden‐Grant et al., *op. cit* note 9; Kerruish and Robertson, *op. cit* note 19; Berg and Powell, *op. cit* note 19; Almond, *op. cit* note 19; Reinstein, *op. cit* note 19; Phadke and Gowda, *op. cit* note 19; Committee on Bioethics, *op. cit* note 23; Grosse et al., *op. cit* note 23; Miller et al., *op. cit* note 25; Clayton, *op. cit* note 28; Dhondt, J. L. (2007). Neonatal screening: From the ‘guthrie age’ to the 'genetic age'. *Journal of Inherited Metabolic Disease*, *30*, 418–422; de Montalembert et al., *op. cit* note 7; Gonzales, J. L. (2011). Ethics for the pediatrician: Genetic testing and newborn screening. *Pediatrics in Review*, *32*, 490–493; Burke, W., Pinsky, L. E., & Press, N. A. (2001). Categorizing genetic tests to identify their ethical, legal, and social implications. *American Journal of Medical Genetics*, *106*, 233–240. 10.1002/ajmg.1001111778984

47
Roberts et al., *op. cit* note 7; Golden‐Grant et al., *op. cit*. note 9.

48
Kerruish and Robertson, *op. cit* note 19; Almond, *op. cit*. note 19.

49
Kerruish and Robertson, *op. cit*. note 19.

50
Almond, *op. cit*. note 19.

51
Roberts et al., *op. cit* note 7; Phadke and Gowda, *op. cit* note 19; Noke et al., *op. cit*. note 37.

52
Botkin and Rothwell, *op. cit*. note 26.

53
Forman et al., *op. cit* note 7; Knoppers et al., *op. cit* note 7; Bailey et al., *op. cit* note 17; Ciarleglio et al., *op. cit* note 19; Berg and Powell, *op. cit* note 19; Arnold, *op. cit* note 26; O'Leary and Maxwell, *op. cit* note 26; Tarini and Goldenberg, *op. cit* note 26; Trotter et al., *op. cit*. note 26.

54
Forman et al., *op. cit*. note 7.

55
Knoppers et al., *op. cit* note 7; Howard et al., *op. cit* note 7; Ross, L. F. (2008). Ethical and policy issues in pediatric genetics. *American Journal of Medical Genetics*, *148C*, 1–7; Kenner, C., & Moran, M. (2005). Newborn screening and genetic testing. *Journal of Midwifery and Womens Health*, *50*, 219–226.

56
Leao and Aguiar, *op. cit* note 7; Clayton, *op. cit* note 28; Bailey, D. (2008). Newborn screening for intellectual disability: Past, present and future. *International Review of Research in Mental Retardation*, *36*, 1–25.

57
Howard et al., *op. cit*. note 7.

58
McCabe and McCabe, *op. cit* note 23; Wieser, *op. cit* note 26; O'Leary and S. Maxwell, *op. cit* note 26; Bonhomme, N. (2013). Population‐based testing: Let's have population‐based discussion. *Genetic Testing and Molecular Biomarkers*, *17*, 265–266. 10.1089/gtmb.2013.154323530538

59
Wilcken, *op. cit* note 7; Knoppers et al., *op. cit* note 7; Howard et al., *op. cit* note 7; Mak et al., *op. cit* note 9; Miller, *op. cit* note 14; Stewart, *op. cit* note 19; Botkin and Rothwell, *op. cit* note 26; Tarini and Goldenberg, *op. cit* note 26; Trotter et al., *op. cit* note 26; DeLuca et al., *op. cit* note 37; Harrell, H. (2009). Currents in contemporary ethics: The role of parents in expanded newborn screening. *Journal of Law, Medicine and Ethics*, *37*, 846–851; Couce, M. L., Macías‐Vidal, J., Castiñeiras, D. E., Bóveda, M. D., Fraga, J. M., Fernández‐Marmiesse, A., & Coll, M. J. (2014). The early detection of Salla disease through second‐tier tests in newborn screening: How to face incidental findings. *European Journal of Medical Genetics*, *57*, 527–531; Fingerhut, R., & Olgemoller, B. (2009). Newborn screening for inborn errors of metabolism and endocrinopathies: An update. *Analytical and Bioanalytical Chemistry*, *393*, 1481–1497; Lantos, J. D. (2016). Introduction to Bioethics special supplement V: Ethical issues in genomic testing of children. *Pediatrics*, *137*, S1–S2; Coman, D., & Bhattacharya, K. (2012). Extended newborn screening: An update for the general paediatrician. *Journal of Paediatrics and Child Health*, *48*, E68–E72. Patch, C. (2006). Newborn screening policy in the United Kingdom & the United States: Two different communities of practice. *MCN. The American Journal of Maternal Child Nursing*, *31*, 164–168. 10.1097/00005721-200605000-0000716679957

60
Khoury et al., *op. cit* note 23; Fingerhut and Olgemoller, *op. cit* note 63; Massie, J., & Gillam, L. (2014). Uncertain diagnosis after newborn screening for cystic fibrosis: An ethics‐based approach to a clinical dilemma. *Pediatric Pulmonology*, *49*, 1–7. 10.1002/ppul.2293324166986

61
Lantos, *op. cit*. note 63.

62
Tarini and Goldenberg, *op. cit*. note 26.

63
Deem, *op. cit*. note 32.

64
There are also other situations in which NBS raises questions about treatment. After screening sometimes one of the parents turns out to have a mild version of a disease, and questions turn up as to whether they need treatment—or not. (Sparks, S. E. (2013). Update on newborn screening*. North Carolina Medical Journal*, *74*, 514–517). 24316780

65
Bunnik et al., *op. cit* note 19; Scala et al., *op. cit* note 19; Deem, *op. cit* note 32; van der Burg, S., & Verweij, M. (2012). Maintaining trust in newborn screening: Compliance and informed consent in the Netherlands. *Hastings Center Report*, *42*, 41–47. 10.1002/hast.6622976412

66
Matsuda, *op. cit* note 7; Saxena, *op. cit* note 7; Spahis and Bowers, *op. cit* note 7; Committee on Bioethics, *op. cit* note 23; Grosse et al., *op. cit* note 23; Atkinson, K., Zuckerman, B., Sharfstein, J. M., Levin, D., Blatt, R. J., & Koh, H. K. (2001). A public health response to emerging technology: Expansion of the Massachusetts newborn screening program. *Public Health Reports*, *116*, 122–131; Clague, A., & Thomas, A. (2002). Neonatal biochemical screening for disease. *Clinica Chimica Acta*, *315*, 99–110. 10.1093/phr/116.2.122PMC149730611847298

67
Baily and Murray, *op. cit* note 7; Elliman, *op. cit* note 7; de Montalembert et al., *op. cit* note 7; Fleischman, A. R., Lin, B. K., & Howse, J. L. (2009). A commentary on the President's Council on bioethics report: The changing moral focus of newborn screening. *Genetics in Medicine*, *11*, 507–509. 10.1097/GIM.0b013e3181a9d66c19478681

68
Golden‐Grant et al., *op. cit* note 9; Moyer et al., *op. cit* note 14; Arnold, *op. cit* note 26; Cragun, D., DeBate, R. D., & Pal, T. (2015). Applying public health screening criteria: How does universal newborn screening compare to universal tumor screening for lynch syndrome in adults with colorectal cancer? *Journal of Genetic Counseling*, *24*, 409–420; Ross, L. F. (2010). Mandatory versus voluntary consent for newborn screening? *Kennedy Institute of Ethics Journal*, *20*, 299–328; Goldenberg, A. J., & Sharp, R. R. (2012). The Ethical hazards and programmatic challenges of genomic newborn screening. *JAMA*, *307*, 461–462.

69
Levy, *op. cit* note 7; Moyer et al., *op. cit* note 14; Tarini and Goldenberg, *op. cit* note 26; Ross, *op. cit*. note 72.

70
Wilcken, *op. cit* note 7; Spahis and Bowers, *op. cit* note 7; Committee on Bioethics, *op. cit* note 23; Therrell, B. L. Jr. (2003). Ethical, legal and social issues in newborn screening in the United States. *Southeast Asian Journal of Tropical Medicine and Public Health*, *34*(Suppl 3), 52–58. 15906695

71
Roberts et al., *op. cit*. note 7.

72
Committee on Bioethics, *op. cit* note 23; van der Burg and Verweij, *op. cit* note 69; Ross, *op. cit*. note 72.

73
Moyer et al., *op. cit* note 14; Committee on Bioethics, *op. cit* note 23; Grosse et al., *op. cit* note 23; Baily et al., *op. cit* note 29; Kenner and Moran, *op. cit* note 58; Ross, *op. cit* note 72; Goldenberg and Sharp, *op. cit* note 72; Larrandaburu, M., Matte, U., Noble, A., Olivera, Z., Sanseverino, M. T., Nacul, L., & Schuler‐Faccini, L. (2015). Ethics, genetics and public policies in Uruguay: Newborn and infant screening as a paradigm. *Journal of Community Genetics*, *6*, 241–249. 10.1007/s12687-015-0236-2PMC452483126021874

74
Dhondt, *op. cit* note 7; Bailey et al., *op. cit*. note 7.

75
Levy, *op. cit* note 7; Knoppers et al., *op. cit* note 7; Bunnik et al., *op. cit* note 19; Berg and Powell, *op. cit* note 19; Khoury et al., *op. cit* note 23; Grosse et al., *op. cit* note 23; Ross, *op. cit* note 46; Harrell, *op. cit* note 63; van der Burg and Verweij, *op. cit* note 69; Ross, *op. cit* note 72; Pelias, M. K., & Markward, N. J. (2001). Newborn screening, informed consent, and future use of archived tissue samples. *Genetic Testing*, *5*, 179–185. 10.1089/1090657015274221811788082

76
Levy., *op. cit* note 7; Knoppers et al., *op. cit*. note 7.

77
Tarini and Goldenberg, *op. cit*. note 26.

78
Ross, *op. cit*. note 72.

79
Ross, *op. cit*. note 72.

80
Bunnik et al., *op. cit* note 19; van der Burg and Verweij, *op. cit* note 69; Ross, *op. cit* note 72; Goldenberg and Sharp, *op. cit* note 72; Sparks, *op. cit*. note 68.

81
Howard et al., *op. cit* note 7; Bunnik et al., *op. cit* note 19; Almond, *op. cit* note 19; DeLuca et al., *op. cit* note 37; de Montalembert et al., *op. cit* note 7; van der Burg and Verweij, *op. cit* note 69; Goldenberg and Sharp, *op. cit*. note 72.

82
Leao and Aguiar, *op. cit* note 7; Miller, *op. cit* note 14; Dhondt, *op. cit* note 47; Giordano, P. C. (2009). Prospective and retrospective primary prevention of hemoglobinopathies in multiethnic societies. *Clinical Biochemistry*, *42*, 1757–1766; Fernhoff, P. M. (2009). Newborn screening for genetic disorders. *Pediatric Clinics of North America*, *56*, 505–513. 10.1016/j.clinbiochem.2009.06.02719591814

83
Kenner and Moran, *op. cit* note 58; Giordano, *op. cit* note 86; Fernhoff, *op. cit* note 86; Ashton‐Prolla, P., Goldim, J. R., Vairo, F. P., da Silveira Matte, U., & Sequeiros, J. (2015). Genomic analysis in the clinic: Benefits and challenges for health care professionals and patients in Brazil. *Journal of Community Genetics*, *6*, 275–283. 10.1007/s12687-015-0238-0PMC452487326040235

84
Elliman, *op. cit* note 7; Dhondt, *op. cit* note 7; Leao and Aguiar, *op. cit* note 7; Bunnik et al., *op. cit* note 19; Stewart, *op. cit* note 19; Almond, *op. cit* note 19; Committee on Bioethics, *op. cit* note 23; Cornel et al., *op. cit* note 23; Tarini and Goldenberg, *op. cit* note 26; Noke et al., *op. cit* note 37; DeLuca et al., *op. cit* note 37; Dhondt, *op. cit* note 47; Fingerhut and Olgemoller, *op. cit* note 63; Ross, *op. cit*. note 72.

85
Roberts et al., *op. cit* note 7; Tarini and Goldenberg, *op. cit* note 26; Noke et al., *op. cit* note 37; Giordano, *op. cit* note 86; Ross, *op. cit*. note 58.

86
Cragun et al., *op. cit*. note 72.

87
Almond, *op. cit*. note 19.

88
Bunnik et al., *op. cit*. note 19.

89
Bailey et al., *op. cit* note 7; Almond, *op. cit* note 19; DeLuca et al., *op. cit* note 37; van der Burg and Verweij, *op. cit*. note 69.

90
Botkin and Rothwell, *op. cit* note 26; Trotter et al., *op. cit*. note 26.

91
Bailey et al., *op. cit* note 7; Almond, *op. cit*. note 19.

92
Howard et al., *op. cit*. note 7.

93

Jennings, B. (2009). Public health and liberty: Beyond the Millian paradigm. Public Health Ethics, 2, 123–134.

94

Mill, J. S. (1859). On liberty (pp. 21–22). Oxford: Oxford University Press.

95
As mentioned before in this review, it is often difficult to make the results of screening evidence based, as the diseases on which it focuses are very rare which makes it difficult to include enough patients situated in similar circumstances in trials. In this discussion, however, including such a disease in NBS seems attractive for further research because this will allow to detect more children with the disease and increases possibilities for research. NBS policy therefore often uses available scientific data as its primary justification, coupled to the prospect of being able to gather more data when the disease is included in NBS.

96
Swierstra and te Molder, *op. cit* note 27; Van der Burg, S., & Swierstra, T. (Eds.) (2013). *Ethics on the laboratory floor*. London/New York: Palgrave Macmillan.

97
Such an approach may draw on the relational approach to public health ethics, as it was suggested by Françoise Baylis, Nuala Kenny and Susan Sherwin (Baylis, F., Kenny, N. P., & Sherwin, S. (2008). A relational account of public health ethics. *Public Health Ethics*, *1*(3), 196–209; Kenny, N. P., Sherwin, S. B., & Baylis, F. E. (2010). Re‐visioning public health ethics: A relational perspective. *Canadian Journal of Public Health*, *101*, 9–11) and by Bruce Jennings (Jennings, B. (2015). Relational liberty revisited: Membership, solidarity and a public health ethics of place. *Public Health Ethics*).

